# Prediction of Visual Memorability with EEG Signals: A Comparative Study †

**DOI:** 10.3390/s20092694

**Published:** 2020-05-09

**Authors:** Sang-Yeong Jo, Jin-Woo Jeong

**Affiliations:** Department of Computer Engineering, Kumoh National Institute of Technology, Gumi 39177, Korea; jsy7057@gmail.com

**Keywords:** visual memorability, electroencephalography, deep learning, machine learning

## Abstract

Visual memorability is a method to measure how easily media contents can be memorized. Predicting the visual memorability of media contents has recently become more important because it can affect the design principles of multimedia visualization, advertisement, etc. Previous studies on the prediction of the visual memorability of images generally exploited visual features (e.g., color intensity and contrast) or semantic information (e.g., class labels) that can be extracted from images. Some other works tried to exploit electroencephalography (EEG) signals of human subjects to predict the memorability of text (e.g., word pairs). Compared to previous works, we focus on predicting the visual memorability of images based on human biological feedback (i.e., EEG signals). For this, we design a visual memory task where each subject is asked to answer whether they correctly remember a particular image 30 min after glancing at a set of images sampled from the LaMemdataset. During the visual memory task, EEG signals are recorded from subjects as human biological feedback. The collected EEG signals are then used to train various classification models for prediction of image memorability. Finally, we evaluate and compare the performance of classification models, including deep convolutional neural networks and classical methods, such as support vector machines, decision trees, and k-nearest neighbors. The experimental results validate that the EEG-based prediction of memorability is still challenging, but a promising approach with various opportunities and potentials.

## 1. Introduction

The memorability of multimedia contents has grown in importance because it can affect people’s decision-making, for example, “to buy a product or not” or “to visit a place again”, and so forth. Therefore, the memorability of contents has been considered as an important issue in the fields of advertisement, visualization, education, etc. In particular, how to measure the memorability of contents is still an open challenge.

**Figure 1:**
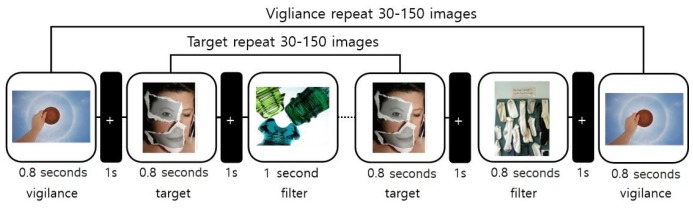
Experimental paradigm of [2].

Previous studies have attempted to determine what contributes to human memory performance. There have been several studies on understanding the memorability of an image with its visual features [1,2,3,4,5]. For example, the authors of [1] tried to determine what makes a visualization more memorable. The memorability of a visualization was measured by a simple memory game, where a set of images was presented sequentially, and the participants were asked to respond whether they remembered a specific image or not. The authors of [1] found that unique visual features tended to have significantly higher memorability scores than common ones. Furthermore, the authors of [2] designed an image memory game similar to that from [1] to measure the memorability of images, as illustrated in Figure 1. As a result of the memory game, a large-scale image dataset containing a set of natural scene images with their corresponding memorability scores was constructed. Based on this dataset, a deep neural network was trained with various levels of visual features to predict the memorability of natural scene images. The authors found that a high-level feature of a scene category plays an important role in predicting the memorability of natural scenes [2]. Similarly, the works in [3,4,5] built their own image memorability databases and generated various kinds of classification models to predict the memorability of images they collected. The work in [5] exploited various visual features with a support vector regression method to predict the memorability of images in different categories. The work from [4] presented a convolutional neural network (CNN) architecture to predict the memorability of a large-scale image database. On the other hand, the work in [3] utilized CNN-generated features and text features for the prediction of the memorability of images.

On the other hand, in the field of neuroscience, it is well known that neural activities occur in a specific area of the brain or specific brain waves are more often observed when an explicit/declarative memory formation is successfully made [6]. With this observation, many researchers in the brain-computer interface (BCI) field have proposed various approaches to memorability prediction. In particular, deep learning approaches, such as CNNs, which have proven to be successful in computer vision tasks, such as object detection and classification, have been applied to the electroencephalography (EEG) domain in recent years. The EEG signal is an electrical voltage captured by electrodes in a portable device attached to the scalp for monitoring brain activities and widely used for BCI systems due to its low cost and non-invasiveness. The studies from [7,8] revealed that there existed a difference in EEG signals between the remembered events and forgotten events. Specifically, Taeho Kang et al. investigated whether it was possible to predict successful memorization of previously-learned words in a language learning context using EEG signals [8]. In their experiments, the participants were asked to memorize German-Korean word pairs, and their retention performance was tested on the same day and the day after learning. The brain signals of each participant were captured using an EEG device and fed into a CNN to predict the memorability of each word pair. Through the experiments, the authors of [8] confirmed that it was possible to predict whether a certain event can be easily memorized or not in a text-based learning context with EEG signals. However, the experimental results with an average accuracy of 62% validated that the EEG-based prediction of memorability in the text domain was still very limited. The work in [9] designed a similar experimental paradigm to conduct a text memory task. With the EEG signals recorded during the memory task, a CNN was trained to classify the EEG signals into remembered and forgotten events. The reported classification accuracy of 72.0% demonstrated that the EEG-based approach to predict the memorability in the text domain was promising, but still had many limitations to overcome.

Recently, multimedia contents, such as images and video resources, have been more extensively consumed, so the memorability of such contents needs to be more studied to further advance applications and services. Compared to the previous studies on predicting the memorability of an image [1,2,3,4,5], we utilized the biological feedback (i.e., EEG) of a human subject as a representative feature to predict the memorability of images. This was based on the assumption that the memorability of an image would also depend on how a user perceives or recognizes the image contents. However, image contents [4] are generally more complex than the text contents used in [8], such as a single word or word pairs, and it can be expected that the prediction of image memorability with EEG signals will be much more challenging. Nevertheless, since images can have unique and distinctive presentations that positively affect the memorability of images [1], analyzing the patterns of biological feedback towards such images will be an interesting issue. Therefore, in this work, we focus on addressing if it is possible to predict the visual memorability of image contents using EEG signals measured from human subjects. For this, we designed a visual memory task consisting of a learning session, where a set of images to remember was provided to the participants, and a test session, where participants’ memory performances were evaluated. The images used in our experiment were sampled from the dataset constructed by [4]. The EEG signals were measured from each subject during the learning session, and then, pre-processed signals were used for various classification methods. In this study, we adopted two networks proposed by [10] as a deep learning-based approach and utilized support vector machines (SVMs), random forests, decision trees, linear regression, and k-NN algorithms as classic machine learning methods. Finally, the analysis of various quantitative and qualitative results from the EEG-based classification of the memorability is presented.

The remainder of this paper is organized as follows. Section 2 describes the details of the proposed experimental protocol and machine/deep learning approaches used in our study. In Section 3, we discuss the experimental results. Finally, we provide the discussions and conclusions in Section 4.

## 2. Method

### 2.1. Experimental Paradigm Design

**Figure 2:**
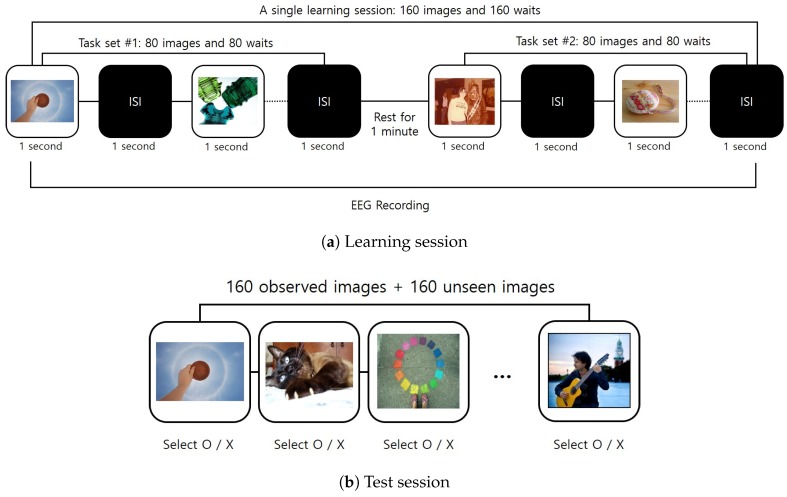
Proposed experimental paradigm.

**Figure 3:**
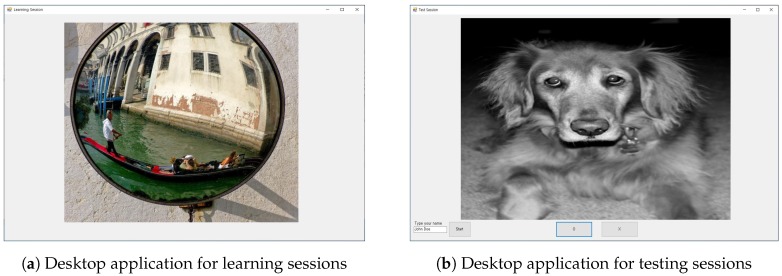
Desktop applications for visual memorability tasks.

We designed an experimental paradigm for a visual memorability task, as illustrated in Figure 2. The proposed visual memory task consisted of learning sessions and testing sessions. In a learning session, a set of images to remember was provided to users in a random order. A testing session evaluated whether a user correctly remembered the images he/she observed during a learning session. As can be seen from Figure 2a, a learning session was composed of two task sets, where a total of 160 randomly sampled images were presented for learning. For each task set in a learning session, each participant was asked to look at the image displayed on a monitor for one second with concentration. There was an inter-stimulus interval (ISI) of one second between the presentation of images, as well as a one minute break between the task sets in a single learning session. The testing session began 30 min after the end of the learning session. In a testing session, the 160 images used in the learning session and another 160 unseen (i.e., new) images were presented in a random order. The subjects are then asked to mark each image as “O”—“I observed this image in the learning session”—or “X”—“I did not see this image in the learning session”—based on their own decision making. Figure 3 presents a snapshot of the desktop application used for learning sessions (Figure 3a) and for testing (Figure 3b).

**Figure 4:**
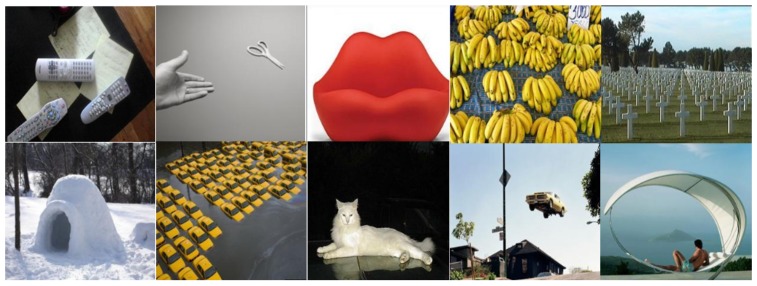
Sample images from the Large-scale Memorability dataset (LaMem) [4].

All of the images used for learning and testing sessions were collected from a Large-scale image Memorability dataset called LaMem [4]. The LaMem dataset includes a variety of image types sampled from existing public image datasets, such as Multimedia Information Retrieval Flickr (MIRFlickr) [11], the affective images dataset [12], and the image popularity dataset [13]. The LaMem dataset contains 60,000 images with a ground-truth memorability score ([0, 1]) for each image obtained using the Amazon Mechanical Turk’s (AMT) microtasks. The memorability score of each image computed from [4] was an indicator of how easily the image could be remembered. Therefore, the lower the value, the lower the likelihood of memorizing the image. As can be seen from Figure 4, the LaMem dataset includes not only scene-centric images, but also object-centric images.

**Figure 5:**
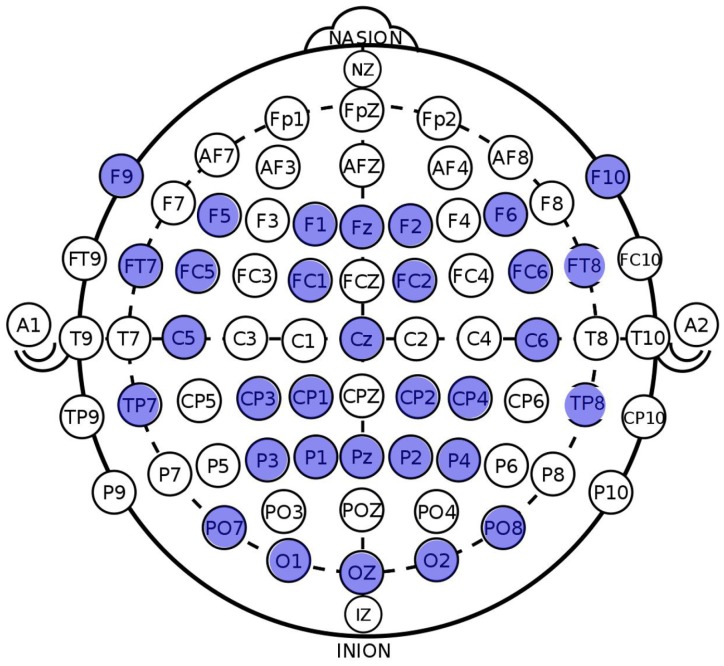
Locations of the EEG electrodes.

Compared to previous works on memorability prediction, we utilized the EEG signals captured during the learning sessions. For recording EEG signals, a 32 channel Emotiv Flex device, an EEG recording equipment manufactured by the EMOTIV company located in San Francisco, USA, with a sampling frequency of 128 Hz and Emotiv Pro software were used. The locations of the electrodes used in our study were customized as F1,2,5,6,9,10,z, FC1,2,5,6, FT7,8, C5,6,z, CP1,2,3,4, TP7,8, P1,2,3,4,z, PO7,8, and O1,2,z under the 10–10 system [14]. Figure 5 illustrates the location of each electrode attached to the scalp. To record EEG signals, each subject was asked to wear an EEG cap during the learning session. After the testing session, the recorded EEG signals for each image were labeled as “remembered” or “forgotten” according to the user responses. The labeled data were used for further training and validation of the machine learning and deep learning networks. For our visual memory experiment, twenty-one voluntary and healthy subjects between 20 and 26 years old (14 males and 7 females) were recruited. The lab environment was strictly controlled to not allow noise and even eye blinking when subjects looked at images. The subjects were asked to perform only one learning session and one testing session per day, but they were supposed to complete two visual memory tasks (i.e., 2 learning sessions + 2 testing sessions), which resulted in exposing them each to 640 images and collecting 320 EEG trials from each subject.

### 2.2. EEG Signal Pre-Processing

Due to the low signal-to-noise ratio (SNR) of EEG signals and the balanced signal power from each location of the electrodes, various restrictions were given to subjects during the EEG acquisition task, and a set of pre-processing steps was usually applied before the analysis of EEG signals.

Similar to [9], the subjects were instructed to minimize their facial, head, arm, and foot movements during signal recording to suppress the influence to EEG recording brought by muscular movements. Furthermore, eye blinking was only allowed during ISIs. In contrast to other EEG-based experiments, a visual memory task designed in this work required that every subject must be given identical stimuli (i.e., images). Failure of this would result in an inconsistent number of trials for each image, which would adversely affect the prediction of the memorability of an image (i.e., computing a memorability score for each image, which will be discussed in Section 3.4). Due to this reason, we could not reject or discard artefactual trials collected from learning sessions. Instead, we conducted a preparatory experiment to inform the subjects of what should be avoided and restricted during the learning session. The movement violation was monitored by an operator [15] during the preparatory experiment, and each subject was given feedback immediately.

As a pre-processing step in this study, bandpass filtering and re-referencing techniques were applied to the collected raw EEG signals. According to previous work [6], both theta and gamma activities were usually identified from healthy participants during declarative memory operations. Therefore, to improve the performance of our memorability prediction, we refined the raw signals with a theta bandpass filter (4–8 Hz) and extracted an EEG segment of 0.3–1 s after onset from each trial. Afterwards, a common average referencing (CAR) method was applied to perform a re-reference procedure, which was done by averaging all the recordings on every electrode. All the pre-processing steps described were performed with the EEGLab MATLAB toolbox [16].

According to [17], almost half of the deep learning-based EEG analysis papers they reviewed (72/154) did not use artifact handling methods. The authors of [17] mentioned that using deep neural networks on EEG might be a way to avoid the explicit artifact removal step of the classical EEG processing pipeline without harming task performance. Inspired by this observation, we minimized the pre-processing step of EEG signals to observe the difference between traditional classification models and deep learning approaches. No other specific artifact removal technique was used except Emotiv built-in features. Instead, we asked each subject to reduce and minimize body movements during the learning session and to have eye blinks during ISIs only as mentioned above.

Similar to [9], the input for each classification model was a matrix *I*, consisting of *N* rows. Each row was a time series of voltage measures with the length of *T*, namely the $d^{\mathrm{th}}$ row was $a_{d}=\left[x_{1},x_{2},\ldots ,x_{T}\right]$. Therefore, the input *I* can be denoted as:

(1) $$I=\left[\begin{array}{c} a_{1}\\ a_{2}\\ \ldots \\ a_{N} \end{array}\right]$$

The size of input *I* was N×T, where *N* is the number of electrodes (channels) and *T* corresponds to the sampling length. In our experiment, *T* was set to be 91 (128 Hz × 0.7 s, representing 0.3–1.0 s). Since we acquired 320 trials for each subject from the learning session, the final data for a single subject consisted of 320 matrices whose size was 32 channels × 91 time-steps. In the other words, all the classification models would take a 32 × 91 matrix as the input of a single trial for each subject.

### 2.3. Classification Models

In this study, we exploited the pre-processed EEG signals as input data to the classification models for predicting the memorability of each image. The classification models used in our study included traditional machine learning algorithms and recent advanced deep learning networks. For the traditional machine learning classifiers, we selected the following methods, which are commonly used in various classification tasks: support vector machines (SVM), logistic regression with stochastic gradient descent training (SLR), decision trees (DT), random forest (RF), and k-nearest neighbors (kNN). For example, an SVM classifier with a filter bank common spatial pattern (FBCSP) method [18] is one of the best performing approaches for motor imagery EEG classification. A brief introduction of each traditional classification model and its corresponding hyper-parameters used in our experiment are as follows. All the mentioned parameters were those that showed the best results during the experiment.

(1) SVM is a supervised learning algorithm that tries to find a n−1-dimensional hyperplane that can divide the *n*-dimensional feature space into two parts. It is well known that the best separation is achieved by the hyper-plane that has the largest distance to the nearest training data points of any class (support vectors). Generally, the larger the margin, the lower the generalization error of the classifier. In our experiment, an RBF-kernel SVM classifier with hyper-parameters C=1 and γ=0.1 was used.
(2) Logistic regression is a probabilistic classifier that makes use of supervised learning. Given a feature vector of a sample, a logistic regression method learns a vector of weights representing how important each input feature is for the classification decision. The probability of a sample is finally computed by a logistic (sigmoid) function. In our experiment, stochastic gradient descent (SGD) with 100 iterations was used as an optimizer, and L2 regularization was applied.
(3) The decision tree observes a set of training samples and extracts a set of decision rules as a tree structure. Decision trees are simple to understand and to interpret; however, they can also generate overly complex trees that do not generalize well, which causes an over-fitting problem. In our experiment, we set the impurity measure for classification to the Gini criterion and the minimum number of samples required for split to 10.
(4) The ensemble method is a technique to combine the predictions from a set of classification models to improve the generalizability or robustness over a single model. The ensemble method of decision trees, the so-called random forest, produces a final prediction value through majority voting for the prediction values from a set of decision trees. In our experiment, the RF classifier used an entropy criterion for the impurity measure, 500 individual trees to make a decision, and the square root of the number of original features as the number of features to consider when searching for the best split.
(5) *K*-nearest neighbor (KNN) algorithm first finds a predefined number (i.e., *k*) of training samples closest in distance to the new sample. Then, the class of a sample is determined by the majority voting over the found nearest neighbors. The distance between the samples is typically measured by the Euclidean metric. In our experiment, the number of neighbors was set to 6.

Over the past few years, the computer vision field has witnessed the great success of deep neural networks for various tasks [19,20,21,22]. The recent advances of deep learning approaches have also resulted in various efforts to design appropriate CNN architectures for decoding EEG signals [10,15,23,24,25,26,27]. Among them, the CNNs presented in [10], called Shallow and Deep ConvNets, which are state-of-the-art networks for the motor imagery EEG classification task, were selected as deep learning-based methods for the prediction of visual memory performance in this study. However, these networks were originally designed for decoding motor imagery EEG signals (e.g., left- or right-hand movement); hence, we optimized a set of parameters and re-trained the models to make them suitable for classification of visual memory performance (i.e., “remembered” or “forgotten”).

**Figure 6:**
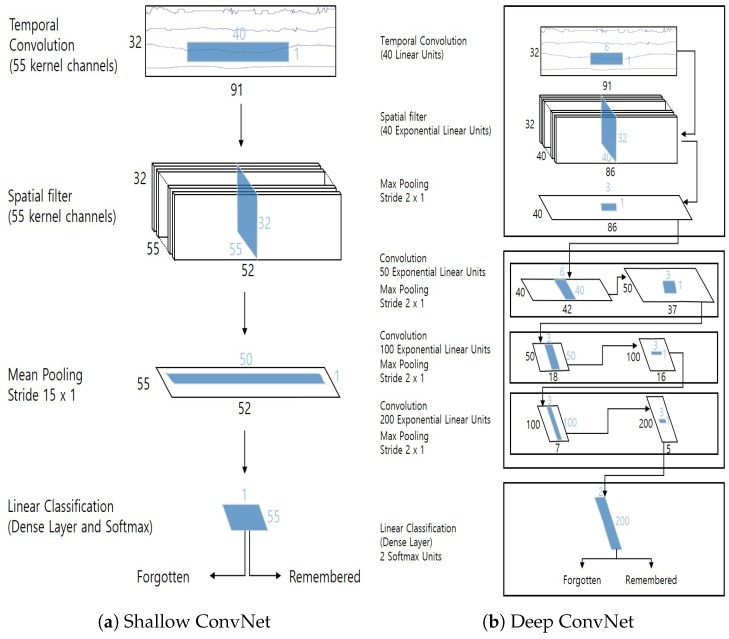
Architectures of Shallow ConvNet and Deep ConvNet.

The architectures of Shallow and Deep ConvNets are depicted in Figure 6a,b, respectively. The first two steps of both Shallow and Deep ConvNets perform temporal and spatial convolution operations. These steps are similar to a bandpass and common spatial filtering step in the filter-bank common spatial pattern (FBCSP) algorithm [28]. After the first two convolution layers, a sequence of additional convolution and/or pooling layers is followed to decode latent features. For Shallow ConvNet, only a single mean pooling layer is used before the final classification. On the other hand, Deep ConvNet exploits much more conv + pool blocks to decode latent features before the classification layer. In the final linear classification layer, a softmax operation is applied to classify EEG signals into the “remembered” or “forgotten” state.

In addition, the AdamWmethod [29] was chosen as our optimizer with a learning rate of 0.0005 and a weight decay of 0.0009 for Shallow ConvNet and a learning rate of 0.008 and a weight decay of 0.0008 for Deep ConvNet. In the case of Deep ConvNet, an exponentially linear unit (ELU) [30] was used as an activation function. Furthermore, to better handle the possible class imbalance problem of input data, we adopted a focal loss [31] function with balanced class weights. The definition of cross-entropy (CE) commonly used for binary classification can be given as:

(2) $$CE\left(p,y\right)=\left\{\begin{array}{cc} -log(p) & if\;y=1\\ -log(1-p) & otherwise \end{array}\right.$$

where *y* is a ground-truth value (e.g., +1/−1) and p∈[0,1] is the model estimated probability for the class with label y=1. Then, CE(p,y) can be rewritten as $CE\left(p_{t}\right)=-log\left(p_{t}\right)$ with the following notation:

(3) $$p_{t}=\left\{\begin{array}{cc} p & if\;y=1\\ 1-p & otherwise \end{array}\right.$$

Finally, the focal loss can be defined as:

(4) $$FL\left(p_{t}\right)=-{\left(1-p_{t}\right)}^{\gamma }log\left(p_{t}\right)$$

As described in Equation (4), focal loss adds a modulating factor (${\left(1-p_{t}\right)}^{\gamma }$) and a tunable focusing parameter γ≥0 as an exponent value into the original cross-entropy (i.e., $-log(p_{t})$ to focus on a class, which is more difficult to predict. According to [31], the modulating factor (${\left(1-p_{t}\right)}^{\gamma }$) reduces the loss contribution from easily classified samples and extends the range in which a sample receives low loss. For example, with γ=2, a sample classified with $p_{t}=0.9$ should have 100× lower loss when compared to CE loss. Therefore, this results in the increase of the importance of correcting misclassified examples. Finally, both ShallowNet and DeepNet were trained until a maximum of epochs of 1000 with an early stopping strategy enabled.

## 3. Experiment

### 3.1. Experimental Setting

The experiment consisted of two phases: (1) visual memory task and (2) memorability prediction task. The goal of the visual memory task illustrated in Figure 2 was to record EEG signals from subjects while they were trying to memorize images. From the visual memory task, we could also have ground-truth data about which image was more easily remembered by subjects. The memorability prediction task was to measure the quantitative (e.g., accuracy) and qualitative performance of classification models using EEG signals. All of the input data for classification models were recorded using the experimental paradigm illustrated in Figure 3a and pre-processed through the protocols described in Section 3.2. The desktop applications used for the learning and testing session to display images and to store user responses were implemented using the C♯ language. All the classic classification models were implemented using the scikit-learn framework [32]. The deep-learning-based classification models were implemented using the BrainDecode framework presented by [10]. Furthermore, the training and validation of all classification models were performed using a desktop PC equipped with an Intel Core i5-7500 CPU, 16GB RAM, and a single NVIDIA GeForce GTX 1080Ti GPU card.

In this section, first we report how users responded during the visual memory task. Then, we present quantitative and qualitative results for the performance of traditional machine learning classifiers and deep learning networks.

### 3.2. User Responses

First, to investigate the reliability of the labeled events (i.e., user responses) from a test session, we computed a matrix for user responses as shown in Table 1. The ratio of user responses of “X” against the unseen images was 93.30% (i.e., 6,270/(450 + 6270)), which implied that the subjects did not maliciously respond during a test session (for example, giving only “O” answers for all test images to argue that they remembered the images very well). Furthermore, the ratios of user responses of “O” and “X” against the seen images were 66.32% and 33.68%, respectively. The hit rate (i.e., the percentage of correct detections by participants) of 66.32% from our experiment was a similar level to that of previous works. The work from [1] reported a hit rate of 55.36% on the visualization images, while [2,5] reported hit rates of 67.5% and 73.7% on natural scene images, respectively. On the other hand, the work in [8] reported a hit rate of 78.7% in a text-based memory task. As described in Section 2, the LaMem dataset [4] used in our experiment consisted of various types of images from object-centric to scene-centric, as well as objects from unconventional viewpoints; therefore, the hit rate of 66.32% measured from our experiment was acceptable, and we could verify the reliability of user responses in that the subjects participated in the experiment with concentration and earnest.

### 3.3. Quantitative Results

In this section, we report the performances of the EEG-based prediction of visual memorability using various classification models including traditional machine learning models and deep neural networks. The evaluation results in this section were measured using 10-fold cross-validation.

First, we show the prediction accuracy of each model, defined as:

(5) $$accuracy=\frac{1}{N}\sum I({\hat{y}}_{i}=y_{i})$$

where *N* is the number of samples, ${\hat{y}}_{i}$ is the predicted value of the *i*^th^ sample, $y_{i}$ is the corresponding true value, and *I* is the indicator function, which returns 1 if the classes match and 0 otherwise.

**Figure 7:**
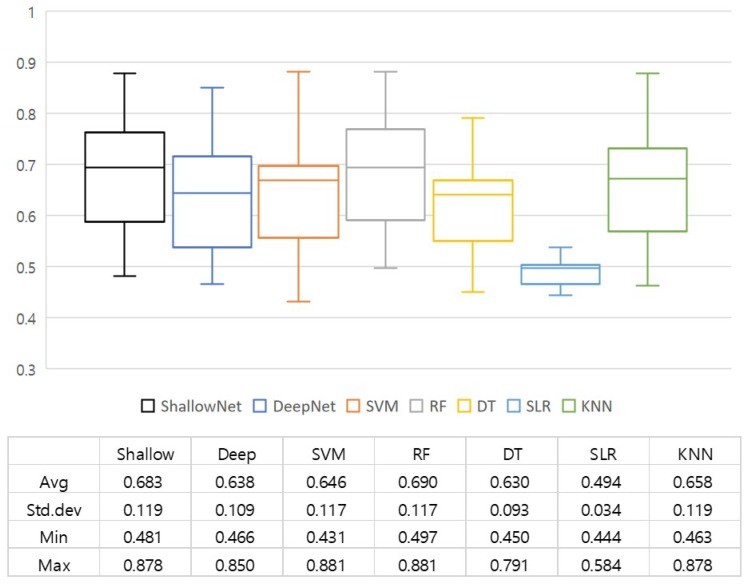
Comparison of accuracy.

Figure 7 presents a summary of the prediction accuracy of each model including average accuracy among subjects, minimum and maximum accuracy, and a standard deviation between accuracy scores of the subjects. It should be noted that there were no significant differences between the performance of deep learning classifiers (Shallow and Deep ConvNets) and that of traditional classifiers. Specifically, all the classification models except SLR produced comparable accuracy, ranging from 0.63 to 0.69 on average. This result was rather different from our expectation that deep-learning-based approaches would produce much better performance than traditional ones. The advantage of deep learning approaches, such as end-to-end learning, automatic feature extraction, and optimization, seemed not to work well for the task to predict the visual memorability of images. This could be due to various reasons including (1) a low SNR of EEG signals, (2) a small number of samples, and (3) inappropriate use of the network configuration. The experimental result described in Figure 7, therefore, confirmed the difficulty of the task addressed in this paper again.

Even though we could not observe a performance improvement from deep learning approaches in our experiment, we could still see how EEG-based classification models worked for the prediction of the visual memorability of images. As shown in Figure 7, among the deep learning-based methods Shallow ConvNet showed the best results in terms of mean accuracy (i.e., 0.683), while Deep ConvNet achieved a slightly lower accuracy (i.e., 0.638). Shallow ConvNet achieved a better performance than Deep ConvNet for most of the subjects. On the other hand, an RF classifier achieved the best results (i.e., 0.69) in terms of mean accuracy among traditional machine learning classifiers. The performance of the RF (0.69) even outperformed the deep learning-based methods (0.683, 0.638). Meanwhile, the SLR classifier showed a relatively lower accuracy (i.e., 0.49 on average) compared to other classification models. The SLR classifiers failed to build a successful classification model, in turn only reaching the level of a random classification (i.e., less than 50% accuracy for a binary classification problem). More details of the accuracy comparison between the methods including a subject-level summary can be found in Table A1. Table 2 presents a summary of the comparison of the accuracy, sensitivity, and specificity between each classification model. Sensitivity measures the proportion of actual positives that are correctly identified as such, while specificity measures the proportion of actual negatives that are correctly identified as such. As can be seen from Table 2, most classification models showed better performance in terms of sensitivity than specificity. This result implied that most classification models were trained to better predict remembered events. This might be due to the imbalance of user responses and each model’s ability to handle this. Future research needs to address this point to achieve better performance.

**Figure 8:**
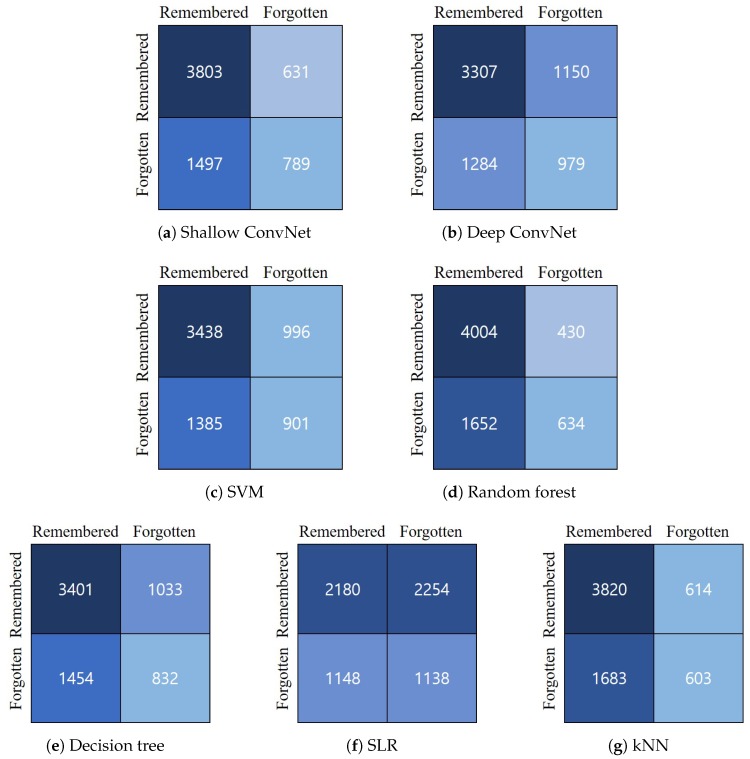
Confusion matrices for each method.

Figure 8 presents a set of confusion matrices for each model. The X-axis represents the class predicted by the classifier, and the Y-axis represents the actual type. The confusion matrices were computed by the sum of all classification results from a 10-fold cross-validation for each subject. As shown in Figure 8, the confusion matrix of each classification model showed a different pattern. The best performing models (i.e., Shallow ConvNet and random forest) had a relatively larger number of true positives for each event. For most classifiers (Shallow ConvNet, SVM, RF, DT, and kNN), we found more false positive cases of remembered events (i.e., predicting “remembered” for actually “forgotten” events) than those of forgotten events (i.e., predicting “forgotten” for actually “remembered” events). These results could be caused by our imbalanced dataset, where the number of data for “remembered” events was twice that for “forgotten” events, so some classifiers could produce slightly biased predictions. Compared to this, Deep ConvNet produced a similar number of false positive cases (i.e., 1150 and 1284) for both remembered and forgotten events and the largest number of true positives for forgotten events (i.e., 979). On the other hand, we could see a confusion matrix of random classification from the result of SLR classifier.

Next, we show the relationship between the behavioral memorability score of our dataset and the pseudo-memorability score predicted by the classifiers. For this, we first computed a behavioral memorability score for each image used in the learning session using the responses from 21 subjects. The behavioral memorability score of a particular image was calculated as the number of subjects who correctly remembered that image divided by the total number subjects (i.e., 21). Because the methods used in this study were not designed to produce a single memorability score, we computed the pseudo-memorability score of a particular image as the number of predictions of “remembered” divided by the total number of subjects. For example, if an image had 17 “remembered” predictions and four “forgotten” predictions, then its pseudo-memorability score was 17/21 = 0.81. To investigate the relationships between the behavioral memorability scores and network-generated pseudo-memorability scores, we measured the Spearman’s rank correlation coefficient (SRCC). As shown in Table 3, the rank correlations between the behavioral memorability scores and pseudo memorability scores from Shallow ConvNet and Deep ConvNet were calculated as 0.7058 and 0.4045, respectively. These results indicated that there existed a high correlation between the pseudo-memorability scores from ShallowNet and the behavioral memorability scores and a moderate correlation between the pseudo-memorability scores from Deep ConvNet and the behavioral memorability scores, respectively. These also implied that the classification models based on the EEG signals had strong potential to predict the memorability of multimedia contents. For comparison, we also computed the SRCC between the pseudo-memorability scores and the ground-truth memorability scores computed by [4]. The rank correlation between the behavioral memorability scores and memorability scores computed by [4] was computed to be 0.5372, which indicated a moderate to high correlation. This value was relatively lower than the human consistency (0.68) on the LaMem dataset computed in [4]. However, this result also came from the difference between the characteristics of the participants (i.e., online anonymous micro-workers (AMT workers) [4] and the offline volunteer students in this work). This resulted in a low to moderate correlation between the pseudo-memorability scores from Shallow/Deep ConvNets and the LaMem memorability scores.

Finally, we summarize the main difference between the proposed and previous works in Table 4. As discussed in the Introduction, most previous works utilized various levels of visual features for prediction and classification of memorability for images. The memorability scores computed in previous works for the image domain showed moderate to high correlations (i.e., 0.31–0.72) with their ground-truth memorability scores. On the other hand, there existed relatively fewer studies focusing on classification and prediction of memorability with EEG signals. Previous works based on EEG signals mainly focused on the text domain (cross-language word pairs or a single word) and produced a moderate level of classification accuracy (i.e., 51–72%). Compared to previous works, we utilized EEG signals for the prediction and classification of memorability for images and then showed the possibility and feasibility of the EEG-based approach by presenting its moderate classification accuracy and correlation values.

### 3.4. Qualitative Analysis

**Figure 9:**
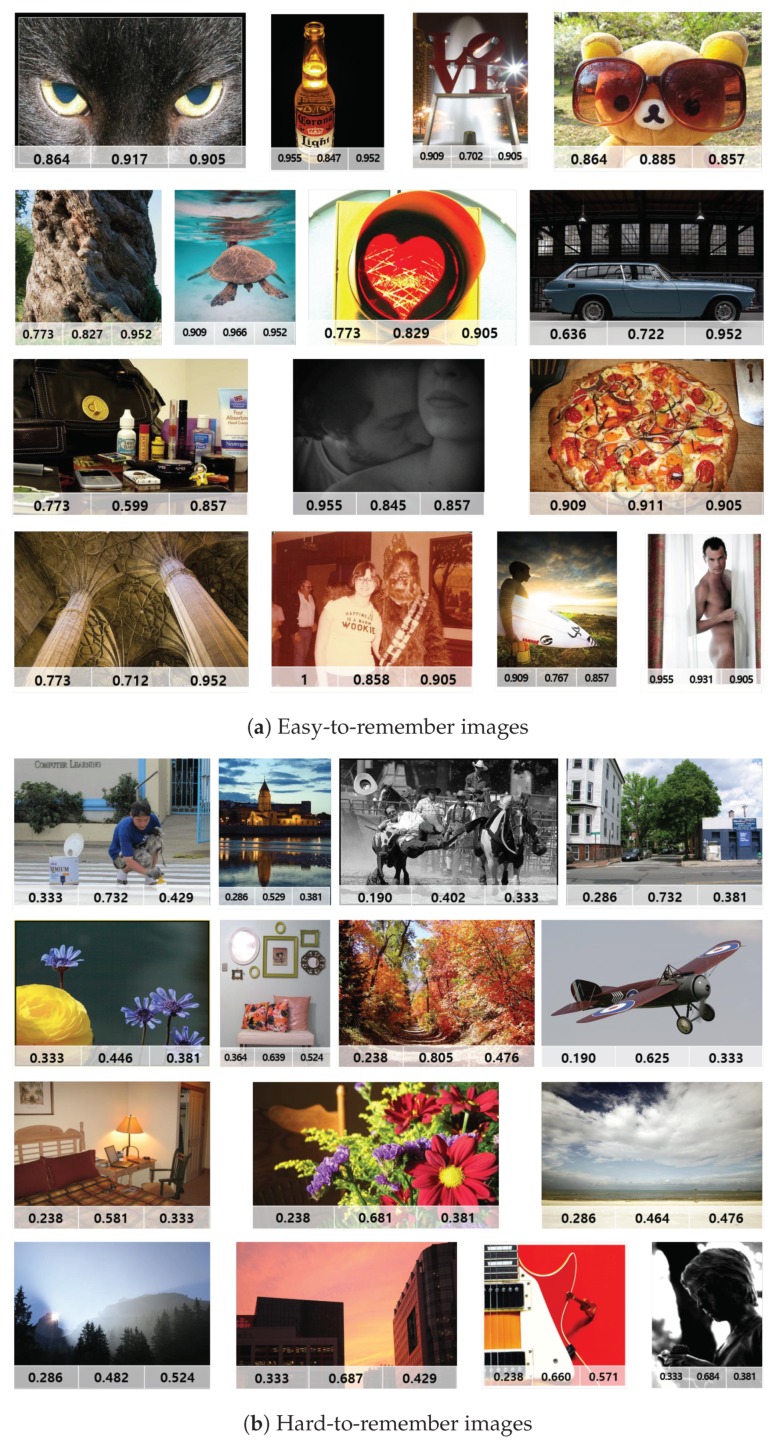
Examples of the top 15 easy-to-remember and hard-to-remember images sorted by pseudo-memorability score from Shallow ConvNet and their corresponding memorability scores (behavioral score, ground-truth score, and pseudo-score, from left to right).

**Figure 10:**
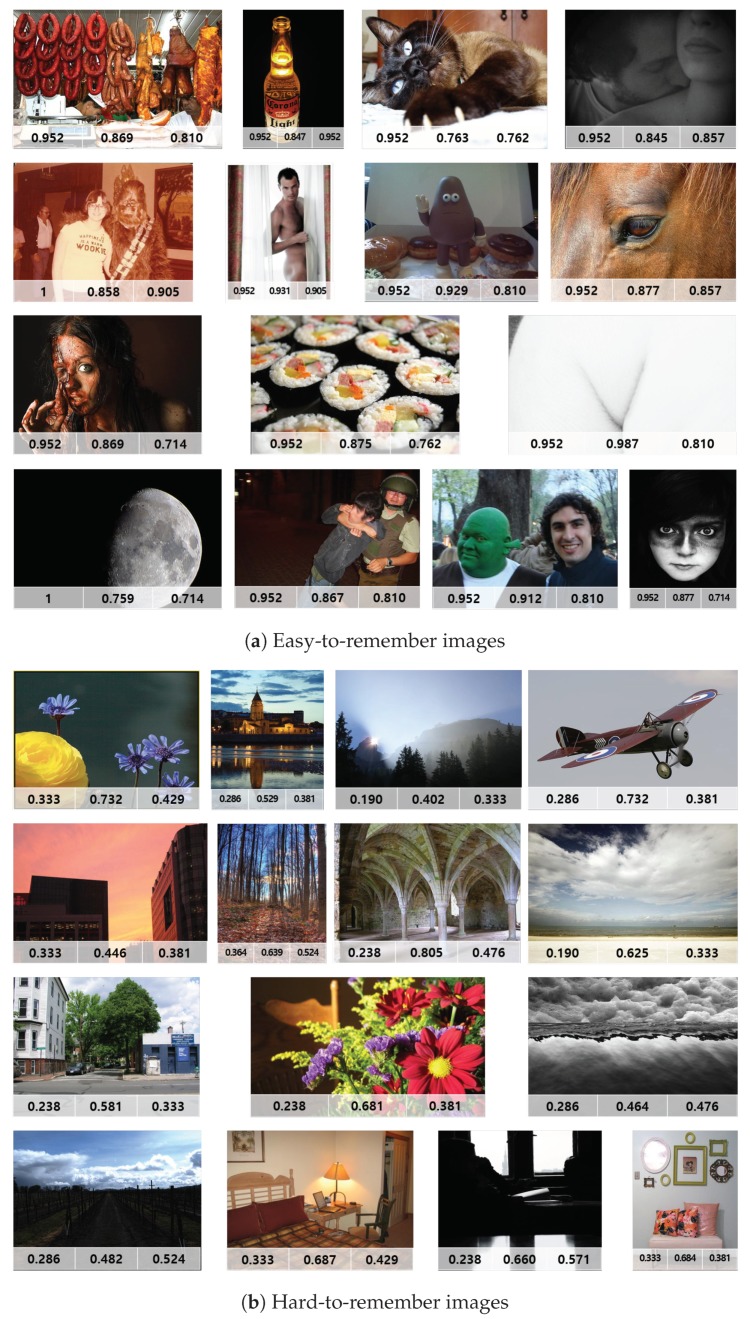
Examples of the top 15 easy-to-remember and hard-to-remember sorted by behavioral memorability score and their corresponding memorability scores (behavioral score, ground-truth score, and pseudo-score, from left to right).

We compared and analyzed the differences between “hard-to-remember” images and “easy-to-remember” images determined by the experimental results. The images in Figure 9 show the top 15 easy-to-remember images and top 15 hard-to-remember images in terms of pseudo-memorability score. The numbers in each image represent the behavioral memorability score, the ground-truth score by [3], and the pseudo-memorability score from the Shallow ConvNet. Similarly, the images in Figure 10 show the top 15 easy-to-remember images and top 15 hard-to-remember images in terms of behavioral memorability score.

First, we investigated the visual difference between easy-to-remember and hard-to-remember images. As shown in Figure 9 and Figure 10, easy-to-remember images tended to have high-level semantic features (i.e., concrete object class), or aesthetically beautiful scenes, or a rare visual appearance. On the other hand, most of the hard-to-remember images tended to have routine and common scenes or uninteresting objects. This confirmed the findings from [2] that a high-level feature of a scene category played an important role in predicting the memorability of a natural scene.

**Figure 11:**
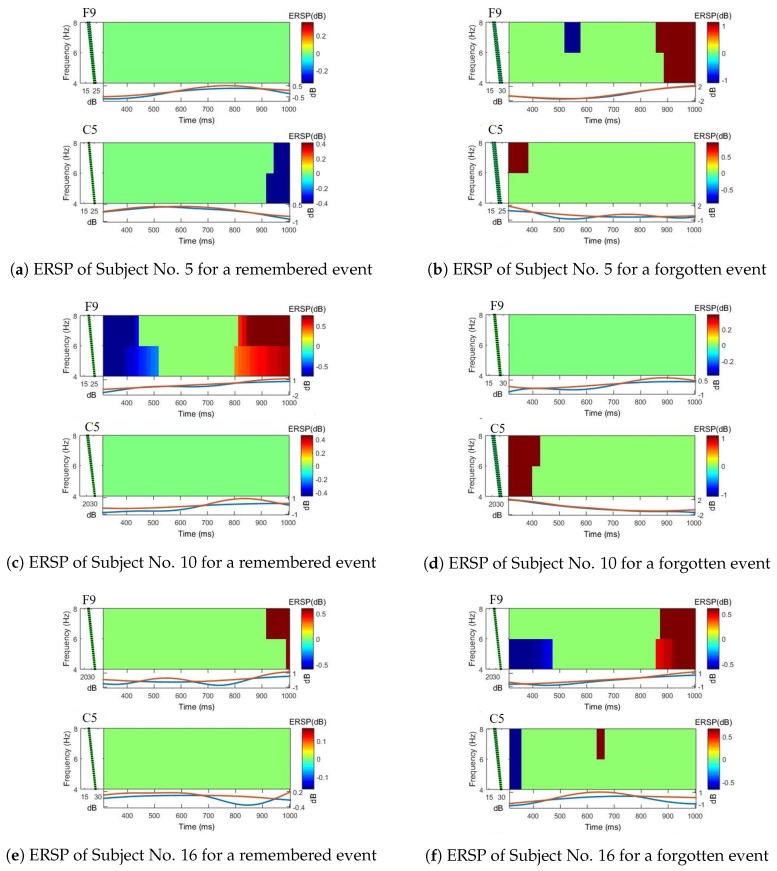
Comparison of the event-related spectral perturbation (ERSP) maps.

Second, we investigated the difference in neural activations between the remembered events and forgotten events during visual memory tasks. For this, we plotted the time-frequency event-related spectral perturbation (ERSP) maps so that the EEG signals were visualized. The ERSP method is to observe the spectral power changes of the induced EEG relative to the stimulus from the view of the time-frequency domain [16]. For *n* trials, if $F_{k}\left(f,t\right)$ is the spectral estimate of trial *k* at frequency *f* and time *t*, the ERSP for *f* and *t* is defined as:

(6) $$ERSP\left(f,t\right)=\frac{1}{n}\sum_{k}^{n}\left(F_{k}{\left(f,t\right)}^{2}\right)$$

where $F_{k}\left(t,f\right)$ can be implemented using the short-time Fourier transform method. The color at each image pixel of the ERSP map then indicated the power at a given frequency and the latency relative to the time locking event [16]. We selected the following three subjects for plotting the map: Subject No. 5, Subject No. 10, and Subject No. 16. The selection was based on the Shallow ConvNet’s classification performance for a subject: (1) high performance group, including Subject No. 5 with an accuracy of 0.86, (2) moderate performance group, including Subject No. 10 with an accuracy of 0.75, and (3) low performance group, including Subject No. 16 with an accuracy of 0.56. Figure 11 provides ERSP maps created from the electrodes F9 and C5 for remembered and forgotten events of each subject. The ERSP map presented here is a plot about the data averaged over all measurements. The selected electrodes (i.e., C5 and F9) to create the ERSP maps were the closest ones to the most contributing electrodes reported in another work [9] on the visual memory task. Finally, Figure 11 reveals that there existed differences in the pattern of neural activations between remembered events and forgotten events. In addition, it was observed that each subject showed a different EEG pattern because the way each subject memorized images was different.

## 4. Conclusions and Discussion

In this study, we exploited EEG signals to predict the memorability of images using various classification models. As discussed in Section 3, it was shown that there existed a difference in EEG signals between remembered events and forgotten events during visual memory tasks. From the experiment with a 10-fold cross-validation, it was found that deep learning-based classification models and traditional machine learning classifiers achieved comparable performances with the best results of 68% from Shallow ConvNet and 69% from the RF classifier. In addition, we found a moderate to high level of SRCC between the pseudo-memorability scores and behavioral memorability scores. Through the experimental results and qualitative analysis, we demonstrated the feasibility of exploiting EEG signals for classification and prediction of memorability for images.

As future work, we will address the following issues. First, the performance of memorability prediction was not stable across subjects. As mentioned above, the low SNR of EEG signals could result in the low performance of classification models. Furthermore, brainwaves are not consistent, even when measured from the same person on the same day, because the physical or mental states of subjects differ. These inherent characteristics of EEG signals lead to inconsistent feature representations for classification models, thereby adversely affecting the overall performance. Our future research will be focused on designing more robust classification models that can handle unstable signals. Furthermore, more EEG data on visual memory tasks will be collected by additional experiments. Second, the visual memory performance of each subject also varied, so that a class imbalance problem occurred for some cases. Therefore, it is expected that exploiting various techniques to handle imbalanced data will improve the performance of classification models. Third, we set 30 min of break time between the learning and testing sessions in the visual memory task. Previous works [4,34] reported that a longer interval tended to decrease the memory performance of subjects. In future experiments, we also plan to investigate the effects of different intervals between the learning and testing sessions on the visual memory performance, as well as classifiers’ prediction results. Finally, we will study a method to better predict the memorability of images in a multi-modal approach (i.e., combination of EEG, EOG, GSR, etc.). 

## Figures and Tables

**Table 1:** Matrix of user responses during test sessions.

	Seen Image	Unseen Image
User response as “O”	4457	450
User response as “X”	2263	6270

**Table 2:** Comparison of accuracy, sensitivity, and specificity.

	Shallow ConvNet	Deep ConvNet	SVM	RF	DT	SLR	KNN
Accuracy	0.68	0.64	0.65	0.69	0.63	0.49	0.66
Sensitivity	0.86	0.74	0.78	0.90	0.77	0.49	0.86
Specificity	0.35	0.43	0.39	0.28	0.36	0.50	0.26

**Table 3:** Comparison of Spearman’s rank correlation coefficient.

	Behavioral Memorability Score	LaMem Memorability Score
ShallowNet pseudo-memorability score	0.71	0.41
DeepNet pseudo-memorability score	0.40	0.20
Behavioral memorability score	-	0.54

**Table 4:** Comparison with other studies.

Work	Domain	Feature	Target	Evaluation	Result
[2]	Image		Memorability score	SRCC	0.58
[4]		Visual features			0.64
[5]					0.31
[3]		CNN feature + caption			0.72
[33]	Image	Visual features	Memorability score	SRCC	0.65
			Memorability classification	Accuracy	82.9
[7]	Text	EEG	Memorability classification	Accuracy	51.18
[8]					61.9
[9]					72.0
**Ours**	**Image**	**EEG**	Memorability score	SRCC	**0.71**
			Memorability classification	Accuracy	**69.0**

## Appendix A. Comparison of Accuracy

**Table A1:** Comparison of accuracy.

	Shallow ConvNet	Deep ConvNet	SVM	RF	DT	SLR	KNN
Subject 1	0.69	0.65	0.69	0.69	0.68	0.49	0.67
Subject 2	0.78	0.77	0.67	0.79	0.77	0.70	0.76
Subject 3	0.55	0.53	0.43	0.59	0.58	0.50	0.46
Subject 4	0.62	0.61	0.61	0.62	0.65	0.54	0.57
Subject 5	0.86	0.73	0.86	0.86	0.70	0.46	0.85
Subject 6	0.70	0.65	0.70	0.69	0.65	0.58	0.66
Subject 7	0.73	0.65	0.72	0.75	0.66	0.50	0.73
Subject 8	0.64	0.64	0.69	0.68	0.62	0.47	0.67
Subject 9	0.75	0.68	0.67	0.75	0.63	0.46	0.70
Subject 10	0.76	0.61	0.71	0.75	0.64	0.44	0.71
Subject 11	0.59	0.58	0.56	0.58	0.55	0.46	0.60
Subject 12	0.89	0.85	0.75	0.88	0.77	0.45	0.88
Subject 13	0.48	0.50	0.48	0.51	0.55	0.47	0.53
Subject 14	0.63	0.60	0.69	0.69	0.55	0.51	0.66
Subject 15	0.54	0.52	0.57	0.56	0.51	0.5	0.47
Subject 16	0.56	0.54	0.57	0.50	0.53	0.49	0.51
Subject 17	0.77	0.76	0.53	0.77	0.67	0.53	0.73
Subject 18	0.88	0.84	0.67	0.88	0.79	0.54	0.78
Subject 19	0.52	0.53	0.88	0.53	0.45	0.49	0.55
Subject 20	0.65	0.47	0.5	0.64	0.56	0.50	0.57
Subject 21	0.76	0.72	0.51	0.78	0.75	0.50	0.77
Min	0.48	0.47	0.43	0.50	0.45	0.44	0.46
Max	0.88	0.85	0.88	0.88	0.79	0.58	0.88
Avg	**0.68**	0.64	0.65	**0.69**	0.63	0.49	0.66
Std. Dev.	0.12	0.11	0.12	0.12	0.09	0.03	0.12
